# Plant manipulation through gall formation constrains amino acid transporter evolution in sap-feeding insects

**DOI:** 10.1186/s12862-017-1000-5

**Published:** 2017-06-27

**Authors:** Chaoyang Zhao, Paul D. Nabity

**Affiliations:** 1 Department of Botany and Plant Sciences, University of California, Riverside, Riverside, CA 92521 USA; 2 Department of Botany and Plant Sciences, University of California, Riverside, 900 University Avenue, Batchelor Hall room 2140, Riverside, CA 92521 USA

**Keywords:** Herbivore, Endosymbiosis, Phylloxeridae, Effector, Aphid, Sternorrhyncha

## Abstract

**Background:**

The herbivore lifestyle leads to encounters with plant toxins and requires mechanisms to overcome suboptimal nutrient availability in plant tissues. Although the evolution of bacterial endosymbiosis alleviated many of these challenges, the ability to manipulate plant nutrient status has evolved in lineages with and without nutritional symbionts. Whether and how these alternative nutrient acquisition strategies interact or constrain insect evolution is unknown. We studied the transcriptomes of galling and free-living aphidomorphs to characterize how amino acid transporter evolution is influenced by the ability to manipulate plant resource availability.

**Results:**

Using a comparative approach we found phylloxerids retain nearly all amino acid transporters as other aphidomorphs, despite loss of nutritional endosymbiosis. Free living species show more transporters than galling species within the same genus, family, or infraorder, indicating plant hosts influence the maintenance and evolution of nutrient transport within herbivores. Transcript profiles also show lineage specificity and suggest some genes may facilitate life without endosymbionts or the galling lifestyle.

**Conclusions:**

The transcript abundance profiles we document across fluid feeding herbivores support plant host constraint on insect amino acid transporter evolution. Given amino acid uptake, transport, and catabolism underlie the success of herbivory as a life history strategy, this suggests that plant host nutrient quality, whether constitutive or induced, alters the selective environment surrounding the evolution and maintenance of endosymbiosis.

**Electronic supplementary material:**

The online version of this article (doi:10.1186/s12862-017-1000-5) contains supplementary material, which is available to authorized users.

## Background

To subsist as an herbivore, an organism must overcome substantial barriers in the form of physical or chemical plant defenses and less than optimal nutrient availability. In some instances, the plant defenses interact directly with nutrient availability by decreasing uptake (e.g., plugged sieve tubes) or impeding digestion (e.g., protease inhibitors), although myriad mechanisms have been described for how herbivores adapt to or avoid defenses [1]. In addition to these deterrents, plant tissues typically maintain high carbon to nitrogen ratios, and plant fluids are depleted in many essential amino acids, making it more difficult for herbivores to acquire nitrogen-based nutrients. To overcome these dietary limitations, herbivores evolved partnerships with bacteria that facilitated transitions to new feeding niches, e.g., on phloem or xylem, or otherwise augmented plant palatability by attenuating defenses [2].

Symbioses can fail, however, when symbiont genomes degrade [3] or limit host range (e.g., plant choice, thermal tolerance; [4]). Thus, there is likely selection pressure to either replace symbionts with more efficient ones [5], or to evolve novel feeding strategies to avoid symbiont dependence. Indeed, several hemipteran lineages, including leafhoppers (Membracoidea: Typhlocybinae) and the Phylloxeridae (Sternorrhyncha: Phylloxeroidea), have transitioned to novel plant-feeding strategies and lost their obligate symbiont associations [4]. The transitions in and out of symbioses have left genomic signatures such as reduced genome structure and function for many obligate symbionts [3, 4], although the effects of symbiosis on herbivore genomes with or without symbionts is unknown.

The metabolic coordination in amino acid synthesis and usage between bacteria and host requires amino acid transporters (AATs) that function in transporting amino acids across the insect/symbiont interface, membranes that separate the cytoplasm of symbionts from insect hemolymph [6]. Two types of AATs mediate this transport: the amino acid polyamine organocation transporter superfamily (APC, transporter classification #2.A.3) and the amino acid/auxin permease transporters family (AAAP, TC #2.A.18). Although both groups belong to the APC superfamily, members of the AAAP family have relatively divergent amino acid sequences, varying substrate specificities, and 11 transmembrane domains, compared to other transporters of the APC family [7, 8]. Expression profiles of these two families of AAT genes for several herbivorous species and their bacterial endosymbionts support a role for these transporters in the evolution of nutritional endosymbiosis [9, 10].

A growing body of evidence has demonstrated that insects induce nutrient sinks in plants in the form of galls that abundantly supply minerals, carbohydrates, and free amino acids [11–16]. Given that numerous insect taxa form galls [17], an intriguing question arises: how does the accessibility of gall-enriched nutritive compounds influence the evolution of insect hosts and/or their symbionts? Among the Sternorrhyncha, few lineages secondarily lost endosymbionts concurrent with a shift to parenchyma feeding [4], and some taxa, such as the Phylloxeridae also induce galls. Insects within the Phylloxeridae are considered sister to the aphids (Aphidoidea: Aphididae) and adelgids (Phylloxeroidea: Adelgidae), groups that also retain galling and free-living species [18]. In contrast with aphids and adelgids that harbor symbionts in bacteriocytes, Phylloxeridae species lack stable intracellular symbionts [19, 20]. Further, Phylloxeridae comprises numerous life history strategies, including galling and free-living species that allow a phylogenetically controlled comparison to understand how these strategies arose with respect to their nutrient acquisition and metabolism. As an important grape pest worldwide, the grape phylloxera (*Daktulosphaira vitifoliae*) is capable of making leaf and root galls and its interaction with plant hosts has been the most investigated among the Phylloxeridae. Studies showed that infestation of *D. vitifoliae* reprograms plant metabolism, leading to the accumulation of nutrients such as carbohydrates and free amino acids [21–23]. Recently, *D. vitifoliae* AATs were compared to paralogs in aphids to help pinpoint which transporters underlie the maintenance of nutrient symbiosis between aphids and *Buchnera* with an emerging conclusion that ecological context may contribute to AAT gene copy number and evolution [10].

To expand the understanding of amino acid metabolism associated with herbivorous insects, we compared species that manipulate plant host amino acid content by gall forming to free-living species, and among species with and without stable nutritional endosymbionts. We sequenced the transcriptomes of nine Phylloxeridae species including *D. vitifoliae* and eight from the genus *Phylloxera.* Oak phylloxera (*P. quercus*) has a free-living life history and thus was compared to other galling phylloxerid species regarding AAT evolution whereas two aphid species whose genomes are sequenced were compared to four other galling aphids. Our results indicated that galling insects, in Phylloxeridae and among aphidomorphs, experienced increased constraints on the evolution of AATs likely because of their ability to manipulate plant host metabolism.

## Methods

### Insect collection

**Fig. 1:**
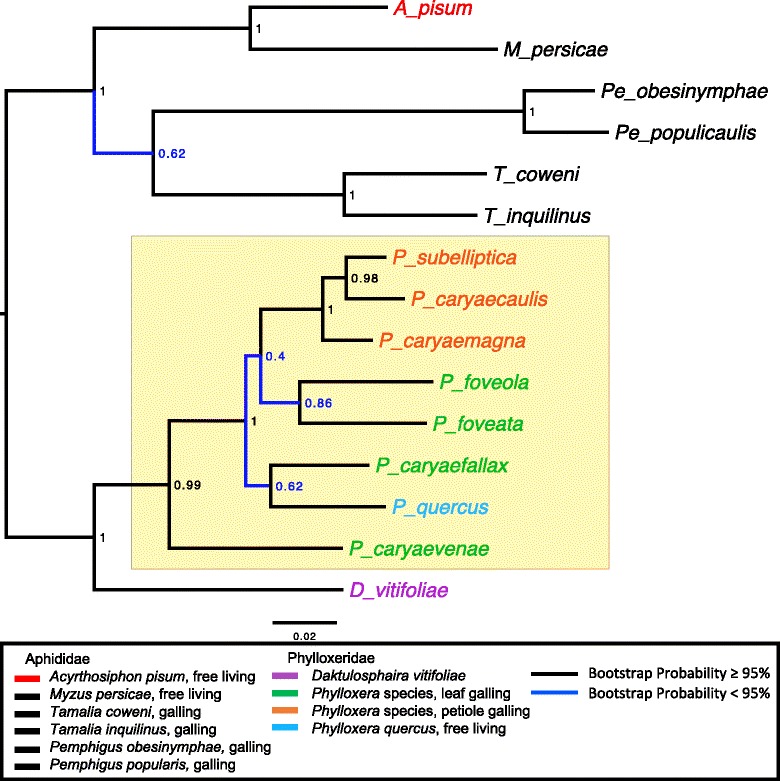
Phylogenetic relatedness of sampled insects within the genus *Phylloxera* (*shaded box*) and related aphidomorphs based on COI, COII, and Cyt b genes. Relatedness support and life history are as indicated in the legend

We collected nine known species within the Phylloxeridae for RNA sequencing. Phylloxeridae is a sister family of Adelgidae under the superfamily Phylloxeroidea, which is sister to the Aphidoidea: Aphididae all within the suborder Sternorrhyncha (Fig. 1). These nine species include three that gall stems/petioles (*Phylloxera caryaecaulis*, *P. subelliptica*, *P. caryaemagna*), one that feeds across hosts causing crinkles/folds in leaf veins (*P. caryaevenae*), three that form spheres on leaves (*P. caryaefallax*, *P. foveata*, *P. foveola*), one freely living (*P. quercus*), and one that galls both roots and leaves of *Vitis* species (*Daktulosphaira vitifoliae*). In contrast to *P. quercus* that lives freely on oak trees (*Quercus spp*.), all the other *Phylloxera* species induce galls on different hickory (*Carya*) species and/or tissues (Table 1). Although the description of *P. foveata* places it on *C. cordiformis* [18] and the individuals collected in this study came from *C. glabra*, we are considering the insects to be the same species for this study because of similarity in the induced phenotype. The Phylloxeridae represents an unresolved taxon [but see 24], where ongoing research is delineating species. All *Carya* originating phylloxerids were collected from the Arnold Arboretum of Harvard University, Boston, Massachusetts. *Phylloxera quercus* was collected from *Quercus sp.* at a horticultural nursery in Bellevue, WA. *Daktulosphaira vitifoliae* was collected from native grapes near Madera Canyon, Arizona. Collected insect samples were stored in RNAlater solution (Qiagen) at room temperature initially, transferred to 4 °C within eight hours for temporal storage (≤ 7 days), and later kept at −80 °C until RNA isolation. Insects at all stages (including eggs) were collected initially but separated to include only juveniles and adults for sequence analysis.

**Table 1:** Number of amino acid transporters (AAT) in sampled insects relative to their life history

				AAT	
Species	Life History	Host	APC	AAAP	Total
Daktulosphaira vitifoliae*	Root & leaf galls	*Vitis*	11	12	23
*Phylloxera caryaecaulis*	Petiole gall	*Carya glabra*	10	16	26
*P. caryaemagna*	Petiole gall	*C. cordiformis*	10	15	25
*P. subelliptica*	Petiole gall	*C. ovata*	10	12	22
*P. caryaevenae*	Leaf gall: fold	*C. glabra*	8	12	20
*P. foveola*	Leaf gall: round	*C. glabra*	7	14	21
*P. foveata*	Leaf gall: round	*C. glabra*	13	17	30
*P. caryaefallax*	Leaf gall: round	*C. ovata*	9	14	23
*P. quercus*	Free-living	*Quercus*	19	24	43
Acyrthosiphon pisum*	Free-living	Fabaceae	18	21	39
Myzus persicae*	Free-living	Diverse	16	20(20)	36
*Pemphigus obesinymphae*	Petiole gall	*Populus*	11(12)	14(14) + 4	29
*Pemphigus populicaulis*	Petiole gall	*Populus*	12	18	30
*Tamalia coweni*	Leaf gall	*Arctostaphylos*	12(9)	16(13) + 5	33
*Tamalia inquilinus*	Inquiline	*Arctostaphylos*	10	17	27
Average	Galling		10	14	24
Average	Free-living		17	21	38
Outgroups: Sternorrhyncha					
*Planococcus citri*	Free-living	Diverse	10	28	38
*Bemisia tabaci*	Free-living	Diverse	12	24	36
*Bactericera cockerelli*	Free-living	Diverse	10	25	28
Outgroup: Non-herbivore					
*Drosophila melanogaster*	Free-living	NA	10	15	25

All gene counts for insects in this study followed the substitution rate method described in the methods except where genomes were available (*). Gene counts are compared to those from [9] show within parentheses, which used a similar gene coalescing method. Some AAAP genes that are expanded in non-arthropods were not reported in previous studies but were in the current study and are designated using “+”. Outgroup Sternorrhyncha herbivores gene counts (from [9]) and non-herbivore *Drosophila melanogaster* are shown

### Transcriptome sequencing and assembly

We used 10–20 individuals for RNA extraction per biological replicate, and performed two replicates for *P. caryaefallax*, *P. subelliptica*, *P. caryaemagna*, *P. caryaecaulis*, *P. quercus* and *D. vitifoliae*, and one replicate for *P. foveata*, *P. foveola* and *P. caryaevenae*. Whole bodies of all insect specimens were homogenized in RTL buffer (Qiagen) and then processed for total RNA extraction using RNeasy Mini kit (Qiagen) following the protocol provided. RNA integrity was examined using a fragment analyzer and samples of the RNA integrity number (RIN) > 7 were used for sequencing. The mRNA library construction and RNA sequencing were performed at the Genomics Core, Washington State University, Spokane, Washington. Briefly, mRNA molecules were enriched using the oligo-dT beads and libraries were constructed for paired-end sequencing on the Illumina HiSeq platform. Raw reads were adaptor-trimmed and filtered to a minimum quality score of 30 over 95% of the read. A single transcriptome reference was generated for each taxon by assembling filtered reads in Trinity with default setting (version 2.1.1; [25]) and assembled sequences were subsequently clustered at a minimum identity of 95% using CD-HIT-EST included in the CD-HIT package (version 4.6.1; [26]).

Raw RNA reads of five Aphididae species, including the green peach aphid (*Myzus persicae*) and four plant-gall related species: *Pemphigus obesinymphae*, *Pe. populicaulis*, *Tamalia coweni* and *T. inquilinus*, were downloaded from the NCBI database (BioProject # PRJNA296778 for *M. persicae*, BioProject # PRJNA301746 for the two *Pemphigus* species, and BioProject # PRJNA297665 for the two *Tamalia* species). Unlike *Pe*. *obesinymphae*, *Pe. populicaulis,* and *T. coweni* that induce galls in plant tissues, *T. inquilinus* does not induce galls but inhabits galls induced by other galling insects [27]. De novo transcriptome references were generated for these five species using Trinity and CD-HIT-EST as described above. The *M. persicae* and draft *D. vitifoliae* genomes available from BIPAA (http://bipaa.genouest.org/is/) were used to compare results from the de novo assembled transcriptomes to help assess how accurate transcript counts were to the true number of annotated genes; however, only *M. persicae* sequences used in this study were taken from the available genome.

### Amino acid transporter annotation

Amino acid transporters in the APC (TC #2.A.3) and AAAP (TC # 2.A.18) families were annotated for all phylloxerid and downloaded aphid sequences following the previously described methods [9, 10, 28]. All bioinformatics tools used here were run at default setting unless explicitly stated. Briefly, longest open reading frames (ORFs) for all transcripts were predicted and translated into protein sequences using a stand-alone PERL script [29]. The protein sequences were searched against the Pfam domain database (Pfam29.0) for functional domains PF03024 (APC) and PF01490 (AAAP) (evalue <0.001) using the HMMSCAN program included in the HMMER software suite (version 3.1b1, [30]). Transcripts with HMMER APC or AAAP hits were verified subsequently by BLAST searching (evalue <0.001) against the NCBI non-redundant protein database. We excluded transcripts derived from possible plant tissue contaminants or other organisms that co-inhabit within the galls induced by Phylloxeridae species, and those of non-APCs or -AAAPs such as Na-K-Cl cotransporters by retaining only transcripts whose best BLAST hits were hemipteran APC or AAAP members.

Because RNA sequencing and assembling approaches assign unique sequence ID for each splicing variant and truncated transcript that are encoded by same gene loci, the identified amino acid transporter transcripts were subsequently collapsed into putative representative loci following the methods previously described [9, 10]. The genomes of *M. persicae* and *D. vitifoliae* were used to map amino acid transporter transcripts to genome scaffold locations using BLASTN searches. Transcripts mapping to the same location were collapsed into the one encoding the longest ORF, or, when partial- or non-overlapping, merged into a single locus. To recover all possible AATs that are encoded by the genomes but were not identified from *M. persicae* and *D. vitifoliae* de novo transcriptome assemblies, we performed BLAST searches (evalue <0.001) using an APC or AAAP transcript of *M. persicae* and *D. vitifoliae*, respectively, against their own genome databases, and those recovered, if any, were subsequently verified at the NCBI non-redundant protein database as described above. For the remaining species lacking draft genome sequences, we: 1) collapsed transcripts having the same Trinity component number into the one encoding the longest ORF, and 2) collapsed closely related transcripts into the one encoding the longest ORF if they have a pairwise synonymous substitution rate (Ks value) less than 0.25 [9] determined using PAML (version 4.8; [31]) or if two transcripts have less than 50-bp of overlapping region, as performed in [10]. All chosen representative transcripts were translated into the longest protein sequences in Blast2GO Pro [32]. Amino acid transporters encoded by *Acyrthosiphon pisum* and *Drosophila melanogaster* genomes were annotated and previously reported [28].

### Phylogenetic analyses

We used DNA sequences of three protein-coding mitochondrial genes, cytochrome c oxidase subunit I (COI), cytochrome c oxidase subunit II (COII) and cytochrome b (CYTB), to resolve the phylogenetic relationship among the nine Phylloxera species and six Aphididae species as described above. COI and COII are widely used to infer insect phylogeny at a variety of hierarchy levels, from closely related species to orders, and CYTB is fast-evolving and thus useful for the phylogenetic analysis of closely-related taxa [33].

The DNA sequences of these three genes were either retrieved from the de novo transcriptomes we assembled or downloaded from the Genbank database (accession # FJ411411.1 for three *A. pisum* genes; accession # NC_029727.1 for three *M. persicae* genes; accession # AM748716.1 for *Pe. obesinymphae* COII). Three gene sequences (COI, COII and CYTB) in each taxon were concatenated to a single one and then aligned using MAFFT (version 7.130) with ‘auto’ setting [34]. The poorly aligned and divergent regions were eliminated on the Gblocks server with default settings [35]. The best-fit nucleotide substitution model was determined in MEGA6 [36], using GTR + G + I. The maximum likelihood method was then run in MEGA6 to construct the phylogenetic trees by testing 1000 bootstrap replications [36].

Phylogenetic analyses of AATs were performed using putative APC protein sequences and AAAP arthropod expanded clade sequences, respectively. AAAP members are composed of the arthropod and non-arthropod expanded clades, between which the sequences are highly divergent [9, 10, 28]. The arthropod expanded clade was so designated because of its multiple gene duplications in the common ancestor of arthropods in contrast to those AAAPs that fall outside this clade [28]. Two *A. pisum* Na-K-Cl transporters (ACYPI001649 and ACYPI007138) and two human SLC36 proteins (SLC36A1 and SLC36A2), which were previously used as outgroups for the phylogenetic analyses of APC and AAAP members, respectively [9, 10, 28], were used likewise in this study. Sequences were aligned using MAFFT with ‘auto’ setting and the alignments were trimmed using TRIMAL (version 1.2) based on a gap threshold of 0.25 [37]. We used MEGA6 to determine the best-fit models of protein evolution, which are LG + G + F for APC proteins and LG + G for AAAP proteins. Because the LG model is not available in the phylogenetics program MRBAYES, we chose the WAG + G + F model for APC and WAG + G for AAAP arthropod expanded clade proteins, and ran the analyses using two runs with 4 chains per run in MRBAYES (version 3.2.1) until the standard deviation of split frequencies between runs dropped below 0.05. The first 25% of generations were discarded and the remaining generations were used to build a 50% majority-rule consensus tree. Lastly, we used the same alignment from above to perform a maximum likelihood inference (RAxML-HPC2) on XSEDE in the CIPRES computing environment [38] for comparison and to generate a consensus topology.

To test if phylogenetically dependent gene families differed per life history for transcript counts within a gene family, we used a PGLS model (counts ~ life history) with a Brownian correlation and a phylogenetic tree using the mitochondrial sequences generated above. Unique AAT sequences were counted for each gene family for each insect used in this study (see Fig. 1) and combined with known counts from three free-living Sternorrhyncha [10] to increase sample size prior to assessing for differences, as a conservative approach. The mitochondrial sequences for the additional insects were obtained from NCBI (NC_030055.1; *Bactericera cockerelli*, KU877168.1; *Bemisia tabaci*, KP692637.1 and AY691419.1; *Planococcus citri*). Sequences were aligned, concatenated using Gblocks to identify conserved mitochondrial sequences, and aligned for a final tree output using RAxML on the CIPRES environment, as described above. The PGLS model was run using the R computing environment and the library ‘picante’ [39].

## Results

Our de novo transcriptome assemblies revealed similar contig numbers and quality metrics as other previously assembled aphidomorph transcriptomes, and among species sequenced for this study (Additional file 1: Table S1). Based upon these assemblies, we present the first multi-locus (CO1, CO11, CYTB) phylogeny across multiple species within the Phylloxeridae (Fig. 1). Our phylogeny indicated phylloxerids first colonized leaves and diversified across host species. Then one lineage evolved to feed on petioles and diversified as it recolonized this tissue across *Carya* species. Thus, species feeding on different tissues of the same plant are likely more distantly related than species feeding on the same tissue of different host species. We also noted that *P. quercus* may represent a unique host switching event given it feeds on oak yet is nested among other hickory feeding *Phylloxera.*

**Fig. 2:**
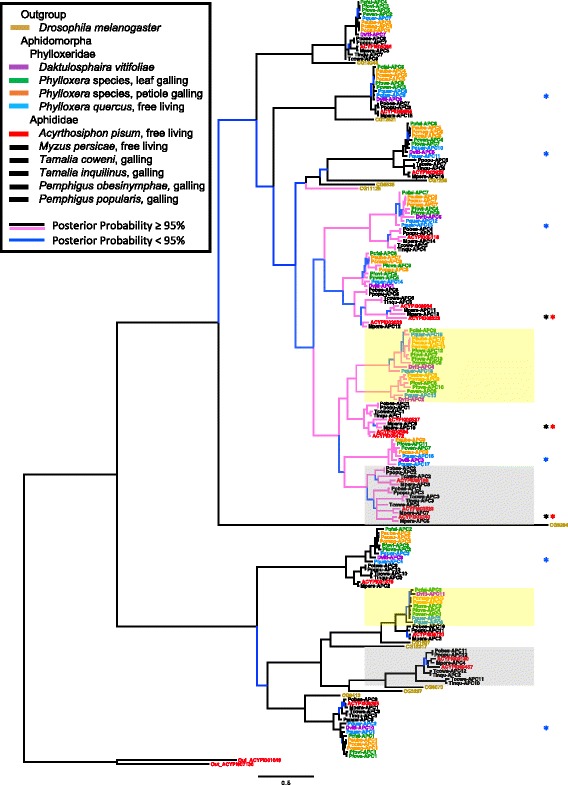
Phylogeny of APC family transporters for sampled insects, representative aphids, and a nonherbivore outgroup. Taxa are color coded per the key, with pink branches denoting the *slimfast* gene expansion shared among aphidomorphs. Shaded regions indicate likely lineage-specific evolution for free-living aphids (*gray boxes*) and phylloxerids (*yellow boxes*). *Asterisks* indicate clades where paralogs occur in free-living aphidomorphs but are lacking in galling species

In comparison with other Sternorrhyncha insects, nearly all phylloxerids retained fewer AATs (Table 1). The one exception is the free-living *P. quercus* where more AATs are abundant than other aphidomorphs and nearly twice the number than in other phylloxerids. Free-living aphids (*A. pisum* and *M. persicae*) also retained more AATs than related gall-feeding aphids in *Pemphigus* or *Tamalia* genera. For APC transporters, gall-feeding insects showed 7–13 (mean = 10) APC transporters whereas free-living aphidomorphs showed more transporters (15–19, mean = 17). Two clades (yellow boxes; Fig. 2) likely contain phylloxerid-specific duplications, where either two genes were present in the phylloxerid ancestor compared to one for the aphid ancestor (upper yellow box), or two phylloxerids (*P. quercus* and *P. foveata*) show duplications compared to a lack of this in aphids (lower yellow box). All other phylloxerid transcripts either strongly clustered with annotated aphid genes, or likely cluster, as indicated by lower bootstrap values. In a comparison of whether life history depended on the number of AATs, both APC and AAAP families showed galling herbivores retained fewer APC and AAAP than free-living species (F = 32.3, *P* < 0.0001); F = 21.4, *P* < 0.0001, respectively). In comparison with genome data, both *M. persicae* and *D. vitifoliae* transcript counts matched the AAT annotated genes with two exceptions for *M. persicae*: 1) two related transcripts mapped to the same gene in the genome, likely caused by an assembly error, rather than indicating a duplication, 2) and two clades where all aphids except *M. persicae* showed gene copies actually had *M. persicae* genes. Thus, the transcript counts were identical to the genome *D. vitifoliae*, and nearly so for *M. persicae*.

**Fig. 3:**
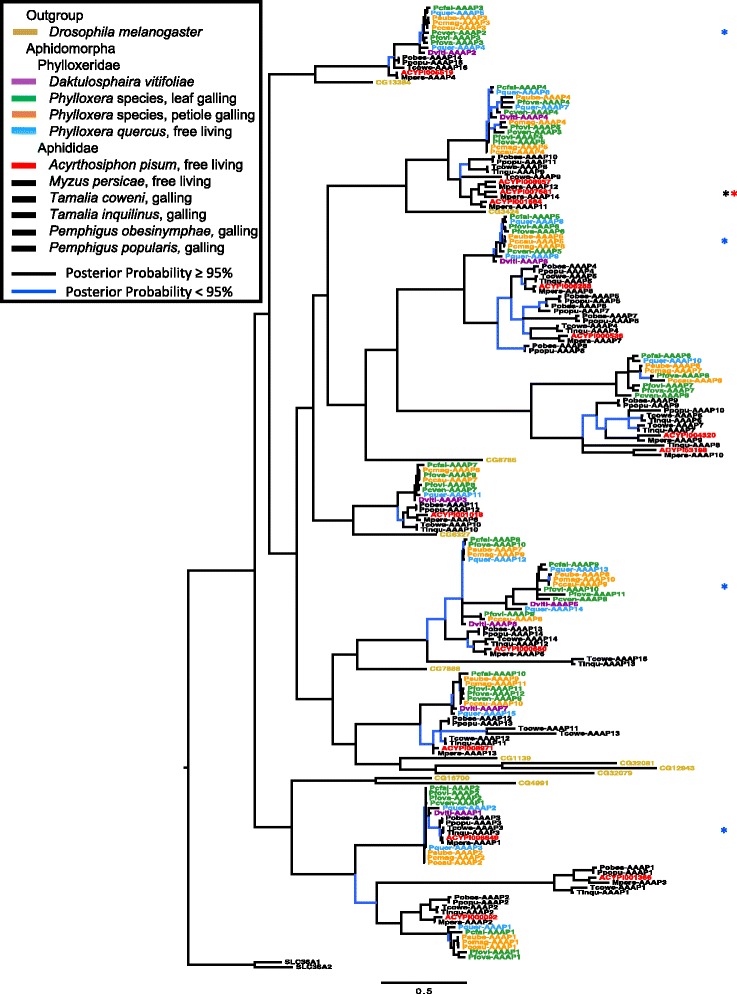
Phylogeny of AAAP family transporters for sampled insects, representative aphids, and a nonherbivore outgroup. Taxa are color coded per the key, with the outgroup human genes SLC1/2 coded in black. *Asterisks* indicate clades where paralogs occur in free-living aphidomorphs but are lacking in galling species

For AAAP transporters, aphidomorphs showed similar abundance profiles, yet have fewer genes than other Sternorrhyncha (see Table 1). However, among aphidomorphs, free-living species showed more AAAP transcripts than galling species, with nearly twice the number in *P. quercus* than other phylloxerids. Among Arthropoda-specific AAAPs, *P. quercus* showed more representative transcripts for seven gene clades whereas free-living aphids differed from galling aphids more variably, with more transcripts in clade two (Additional file 1: Table S2, Fig. 3). Some lineage specificity occurred with *Pemphigus* showing more AAAPs in clade three and *Tamalia* more in clade four. Among non-arthropod genes free-living aphidomorphs show more transcripts than galling aphidomorphs across clades (Additional file 1: Table S2).

## Discussion

Amino acid uptake, transport, and catabolism underlie the success of herbivory as a life history strategy [29, 40]. Here we present the first multigene tree for members within the Phylloxeridae; a family with both galling and free-living herbivores. We also present transcript profiles across fluid feeding herbivores that support plant host constraint on insect amino acid transporter evolution. Galling sap-feeding insects show fewer AAT transcripts than free-living species within the same insect families and within the same genus of *Phylloxera*. The ability of galling insects to manipulate plant nutrient content likely altered selection to retain or duplicate the number of functioning AATs within the insect. Previous research suggests some AATs facilitate the evolution of endosymbioses but also that ecological context may interact with nutrient transporter evolution to shape adaptive duplication or loss [10]. Our data advance this idea by highlighting how complex the selective environment is and suggest specialized interactions with plants play a large role in determining the evolution of herbivore genomes, especially when nutrient manipulating strategies are involved.

Previous research on some AATs correlates gene expression and presence with the maintenance of endosymbioses; however, phylloxerids lack stable endosymbionts and still retain many of these same AATs. We found members of the Phylloxeridae family retain at least one copy of each APC found among other aphidomorphs (as in [10]) with the exception of two clades (yellow boxes; Fig. 2) that show duplications. Otherwise, phylloxerids retained at least one APC similar to many other Sternorrhyncha insects and *D. melanogaster* [28]. Interestingly, free-living *P. quercus* often showed multiple AAT copies within clades where galling phylloxerids possessed only one copy (blue asterisks; Fig. 2). Free-living aphids also show a similar pattern compared to galling aphids for many clades (black and red asterisks; Fig. 2). This increase within clades suggests that these paralogs may function generally to support nutrient transport when feeding on host parenchyma, a tissue where nutrients are lower than when feeding on gall tissue where nutrients can be enriched by and for the galling insect. We hypothesize then that host nutrient manipulation altered the selection environment to maintain certain AATs. In support of this we identified fewer AATs in galling insects than free-living relatives. In some instances, galling phylloxerids did not accumulate specific AAT transcripts; however, lack of accumulation may result from a missing gene or lack of conditions under which expression occurs. While we recognize the limitations of transcriptome information to resolve this, the use of the *D. vitifoliae* and *M. persicae* genomes suggests all phylloxerid and nearly all aphid genes were accounted for, and that variation in AATs among genera occurs within aphidomorphs. Some *Phylloxera* species show accumulation of AATs absent from the *D. vitifoliae* genome whereas galling *Phylloxera* species show different numbers of AAT transcripts across clades. Similarly, some galling aphids (e.g., *T. coweni*) also lack transcripts for some clades where free-living aphids retain one if not more transcripts. This provides support that differences in the nutrient environments across plant hosts differentially alter selection to retain certain AATs. Little information exists for comparing extensive metabolite profiles of hosts across galled taxa, but the diversity in morphology, color, and specialized tissues that are induced in plant hosts by galling insects [41] suggests nutrient pools that insects feed upon differ widely. Future studies examining metabolite pools among closely related taxa will help resolve what limitations, if any, are present in induced plant phenotypes, and provide additional tests of the role of host nutrient manipulation in the evolution of insect AATs.

The microbial community plays a fundamental role in animal nutrient acquisition from food, especially for sap-feeding insects where coevolution with endosymbiotic bacteria alleviates low amino acid content provided by phloem diets [42]. Galling or less apparent manipulation of host nutrients (e.g., delayed host senescence by leaf miners; [43]) increases nutrient flux to feeding sites, potentially altering selection on the stability of endosymbiotic relationships. Our data and previous transcriptome profiles support increases in paralogs for two free-living aphids, but no galling aphids share these increases. This pattern suggests lineage specificity [10]; however, until more insects are profiled in a way that controls for phylogeny while spanning the range of plant nutrient manipulation, either host manipulation, lineage specific evolution, or both may alter selection on AAT gene evolution.

Prior transcriptome assessments [9, 10] correlated transcript abundance and presence with maintaining endosymbiosis. By examining more phylloxerids, we increased resolution of *slimfast* gene evolution, providing support for previous data that all aphidomorphs experienced *slimfast* duplication (Fig. 2). Thus, *slimfast* expansions likely occurred in the ancestral aphidomorph. Because the ancestral state of phylloxerids is unresolved without a phylogeny, it is possible that the ancestor lost AATs with the evolution of galling or gained AATs with the transition away from galling. Either scenario would link this clade to nutrient acquisition strategies. Although phylloxerids lack stable nutritional endosymbionts, numerous microbial partners exist within the gut microbiome (PDN unpublished data, [20]). How gut microbial community dynamics modify sap-feeder fitness is less well understood. Profiling partner presence and abundance alongside plant host nutrients may highlight specific roles, if any, for microbes in an insect that lost endosymbionts.

The patterns in AAT transcripts within the Phylloxeridae, and among galling and free-living Aphidomorpha, provide insight into the selection imparted through host manipulation and the evolution (or loss) of endosymbiosis. *Slimfast* paralogs appear across many phylloxerids, and thus appear not to drive endosymbiosis [10]. Rather the presence of *slimfast* duplications in free-living *Phylloxera* suggest an additional role in nutrient transport when the host cannot be manipulated. Although our data support the *slimfast* expansion among aphidomorphs, we hypothesize that plant host nutrient availability may have facilitated some duplications found in free-living pea and green peach aphids because several paralogs appear absent from galling aphids. Recent cellular localization screens indicated one of these genes (ACYPI008904) increases in expression prior to and after *Buchnera* transmission as the bacteriocyte develops [44]. Because galling aphids lack ACYPI008904 paralogs, selection to duplicate specific nutrient transporters may be relaxed when plant host nutrient status can be manipulated. Notably, the emergence of phylloxerid-specific duplications (yellow shading; Fig. 2) related to these aphid genes, highlights a bacteriocyte-independent role for some *slimfast* orthologs.

## Conclusion

Transcriptional profiling of AATs across Sternorrhyncha insects has revealed patterns of co-expression between symbiont and host, and identified gene candidates that may underlie the maintenance and evolution of endosymbiosis. While lineages that contain stable nutritional symbionts provide a model for dissecting how gene expression correlates with symbiosis, insect relatives that lack nutritional endosymbionts provide additional context on how symbioses can evolve or what may lead to losses in symbionts. Here we show that phylloxerids retain many of the AATs of other symbiont-harboring aphidomorphs, and that a pattern is emerging for plant manipulation to constrain AAT evolution. How insects alter host-plant phenotypes, whether chemical or morphological, is largely unknown. Growing evidence suggests that secreted peptides called effectors underlie these induced changes [45], and may target immune function to enable colonization, but also regulate fundamental development pathways that coordinate nutrient transport [46, 47]. Going forward, it is likely that the evolution of plant manipulation interacts with the maintenance of symbiosis to perturb host-symbiont relationships. Understanding genomic patterns in effector families, nutritional symbionts, and host-plant nutrient allocation within a phylogenetic context may provide key insight into the evolution of insect nutrient acquisition, whether through a symbiont, microbe derived effector, or de novo effector evolution to target conserved plant signaling or nutrient mobilization networks. Undoubtedly, the evolution of herbivory is complex, but comparative studies across taxa will continue to provide context on processes, ecological or otherwise, that facilitated and maintain herbivory.

## Additional file

Additional file 1: Tables S1. Quality control and assembly statistics for transcriptomes used in analyses. **Tables S2.** Mitochondrial gene sequences used to generate species-level phylogeny of insects assessed in this study. **Tables S3.** AAT gene sequences for APC and AAAP gene families for each species assessed in this study. **Tables S4.** Summary of AAT gene counts per clade for each species assessed in this study. **Tables S5.** Summary of dS values or overlapping states used to merge transcripts for APC genes. **Tables S6.** Summary of dS values or overlapping states used to merge transcripts for AAAP genes. (XLSX 204 kb)

## Abbreviations

AAAP: Amino acid/auxin permease transporter
AAT: Amino acid transporter
APC: Amino acid polyamine organocation transporter
COI: Cytochrome oxidase I
COII: Cytochrome oxidase II
CYTB: Cytochrome b
